# Neuronal over-expression of Oxr1 is protective against ALS-associated mutant TDP-43 mislocalisation in motor neurons and neuromuscular defects *in vivo*

**DOI:** 10.1093/hmg/ddz190

**Published:** 2019-09-06

**Authors:** Matthew G Williamson, Mattéa J Finelli, James N Sleigh, Amy Reddington, David Gordon, Kevin Talbot, Kay E Davies, Peter L Oliver

**Affiliations:** 1 Department of Physiology, Anatomy and Genetics, University of Oxford, Parks Road, Oxford OX1 3PT, UK; 2 Department of Neuromuscular Diseases, Institute of Neurology, University College London, London WC1N 3BG, UK; 3 UK Dementia Research Institute, University College London, London WC1E 6BT, UK; 4 Nuffield Department of Clinical Neurosciences, University of Oxford, West Wing, John Radcliffe Hospital, Oxford OX3 9DU, UK; 5 MRC Harwell Institute, Harwell Campus, Didcot, Oxfordshire, OX11 0RD, UK

## Abstract

A common pathological hallmark of amyotrophic lateral sclerosis (ALS) and the related neurodegenerative disorder frontotemporal dementia, is the cellular mislocalization of transactive response DNA-binding protein 43 kDa (TDP-43). Additionally, multiple mutations in the *TARDBP* gene (encoding TDP-43) are associated with familial forms of ALS. While the exact role for TDP-43 in the onset and progression of ALS remains unclear, the identification of factors that can prevent aberrant TDP-43 localization and function could be clinically beneficial. Previously, we discovered that the oxidation resistance 1 (Oxr1) protein could alleviate cellular mislocalization phenotypes associated with TDP-43 mutations, and that over-expression of Oxr1 was able to delay neuromuscular abnormalities in the hSOD1^G93A^ ALS mouse model. Here, to determine whether Oxr1 can protect against TDP-43-associated phenotypes *in vitro* and *in vivo*, we used the same genetic approach in a newly described transgenic mouse expressing the human TDP-43 locus harbouring an ALS disease mutation (TDP-43^M337V^). We show in primary motor neurons from TDP-43^M337V^ mice that genetically-driven Oxr1 over-expression significantly alleviates cytoplasmic mislocalization of mutant TDP-43. We also further quantified newly-identified, late-onset neuromuscular phenotypes of this mutant line, and demonstrate that neuronal Oxr1 over-expression causes a significant reduction in muscle denervation and neuromuscular junction degeneration in homozygous mutants in parallel with improved motor function and a reduction in neuroinflammation. Together these data support the application of Oxr1 as a viable and safe modifier of TDP-43-associated ALS phenotypes.

## Introduction

Amyotrophic lateral sclerosis (ALS) is characterized by the progressive loss of upper motor neurons in the cerebral cortex and lower motor neurons in the brainstem and spinal cord, leading to severe muscle wasting and death due to respiratory failure (1). ALS is now understood to have a complex etiology, with mutations in more than 20 genes associated with both familial and some apparently sporadic forms; however, no identified genetic contribution has been discovered in the majority (~85%) of cases. Despite this heterogeneity, over 95% of all ALS cases share the pathological signature of mislocalized TDP-43 (43-kDa transactive response DNA-binding protein) (2,3). Typically, cytoplasmic aggregates containing hyper-phosphorylated and ubiquitinated TDP-43 protein are found in affected motor neurons in ALS, and in frontotemporal dementia (FTD), forming a disease spectrum known as the TDP-43 proteinopathies (4). In addition, mutations in the *TARDBP* gene, encoding TDP-43, account for ~5% of familial ALS (fALS), <1% of sporadic ALS, as well as rare familial cases of FTD (5–8). To date, genetic studies have revealed over 50 mutations in TDP-43 (9) and the majority are clustered in the glycine-rich C-terminal domain (10).

TDP-43 is a ubiquitously expressed DNA/RNA-binding protein that functions in a wide range of cellular processes including transcriptional regulation, RNA splicing and micro-RNA biogenesis (11,12). TDP-43 is predominantly found in the nucleus, however, under conditions of cellular stress, such as oxidative stress observed in ALS, TDP-43 relocalizes to the cytoplasm where it facilitates the formation and dissociation of stress granules (13–15). Whether the process of TDP-43 mislocalization is itself required for the onset or progression of ALS is still debated. Therefore, a complex picture is emerging whereby loss of normal nuclear function may act in combination with aberrant cytoplasmic toxicity. As such, although the molecular mechanisms underlying the role TDP-43 in ALS are still under investigation, factors that are able to alleviate the mislocalization and/or other aberrant roles of TDP-43 mutants in neuromuscular function may provide therapeutic potential.

Cellular studies have begun to reveal the properties of individual TDP-43 mutations, with over-expression typically driving reproducible cytoplasmic mislocalization, thus promoting the search for modifiers of this fundamental feature of TDP-43 biology *in vitro* (16). For instance, oxidation resistance 1 (OXR1) is a member of a family of proteins containing the TLDc domain that is protective against oxidative stress-related insults (17–19). During a screen for protein interactors in neuronal cells, we identified TDP-43 as an Oxr1 binding partner (20). Interestingly, over-expression of a short functional isoform of Oxr1 (Oxr1-C)—consisting of almost exclusively the TLDc domain—reduced cytoplasmic mislocalization and aggregation of TDP-43 mutants where binding to Oxr1 was preserved (M337V and Q331K), suggesting that Oxr1 has the potential to alleviate TDP-43-associated pathology such as mitochondrial dysfunction and oxidative stress-induced apoptosis (20). Further evidence that Oxr1 is protective against ALS-associated phenotypes was revealed during an *in vivo* study of the hSOD1^G93A^ mouse model, where neuronal over-expression of Oxr1 extended survival of mutants while significantly delaying spinal cord and muscle pathology, including reducing neuroinflammation (21). Crucially, over-expression of Oxr1 was not detrimental to the mice, indicating that Oxr1 could be a positive candidate for the modification of mutant TDP-43 function *in vivo* (21).

To date, a range of rodent models expressing TDP-43 mutations has been generated in an attempt to understand the pathobiology of TDP-43 dysfunction. In general, mouse models that have utilized expression of a TDP-43 transgene have recapitulated certain ALS-associated phenotypes such as motor dysfunction, although toxicity driven by artificial promoters generating non-physiological levels of the protein likely explains the variable severity of the pathologies observed (22–31). For example, mice over-expressing neuronal TDP-43 containing the ALS-associated M337V mutation show severe motor neuron loss, neuromuscular junction (NMJ) degeneration and premature death by 1 month of age (30,31), whereas mice hemizygous for the same transgene display a far-less severe motor phenotype (32). In order to circumvent the inherent limitations of these approaches, mice with a single copy of either a wild-type (WT) or mutant M337V human bacterial artificial chromosome (BAC) containing the entire TDP-43 locus were generated recently (16,33). Expression of TDP-43^M337V^ from the native human promoter leads to progressive motor impairment and disruption of the NMJ from 6 months of age, thus facilitating longitudinal studies of the pre- and post-symptomatic stages of pathogenesis (33); importantly, mice expressing the identical WT BAC (TDP-43^WT^) display no pathological or behavioural abnormalities at any timepoint, demonstrating the specific pathological effects of the mutant transgene (33).

Here, given that Oxr1 over-expression can delay pathogenesis in the hSOD1^G93A^ model (21) and reduce mislocalization and aggregation of mutant TDP-43^M337V^*in vitro* (20), we aimed to investigate the neuroprotective function of Oxr1 in the context of *in vivo* expression of TDP-43^M337V^ in this mouse model. First, we demonstrate that cytoplasmic mislocalisation of mutant TDP-43 in primary motor neurons from TDP-43^M337V^ mutants is alleviated by Oxr1 over-expression. We go on to show that neuronal over-expression of Oxr1 significantly improves motor function as well as newly-characterized neuromuscular, muscular and neuroinflammatory phenotypes in TDP-43^M337V^ mice. Together these data demonstrate the promise of Oxr1 as a valuable modifier of TDP-43-associated ALS phenotypes.

## Results

### Genetic over-expression of Oxr1 reduces aberrant TDP-43 mislocalization in primary motor neurons from TDP-43^M337V/−^ mice

**Figure 1:**
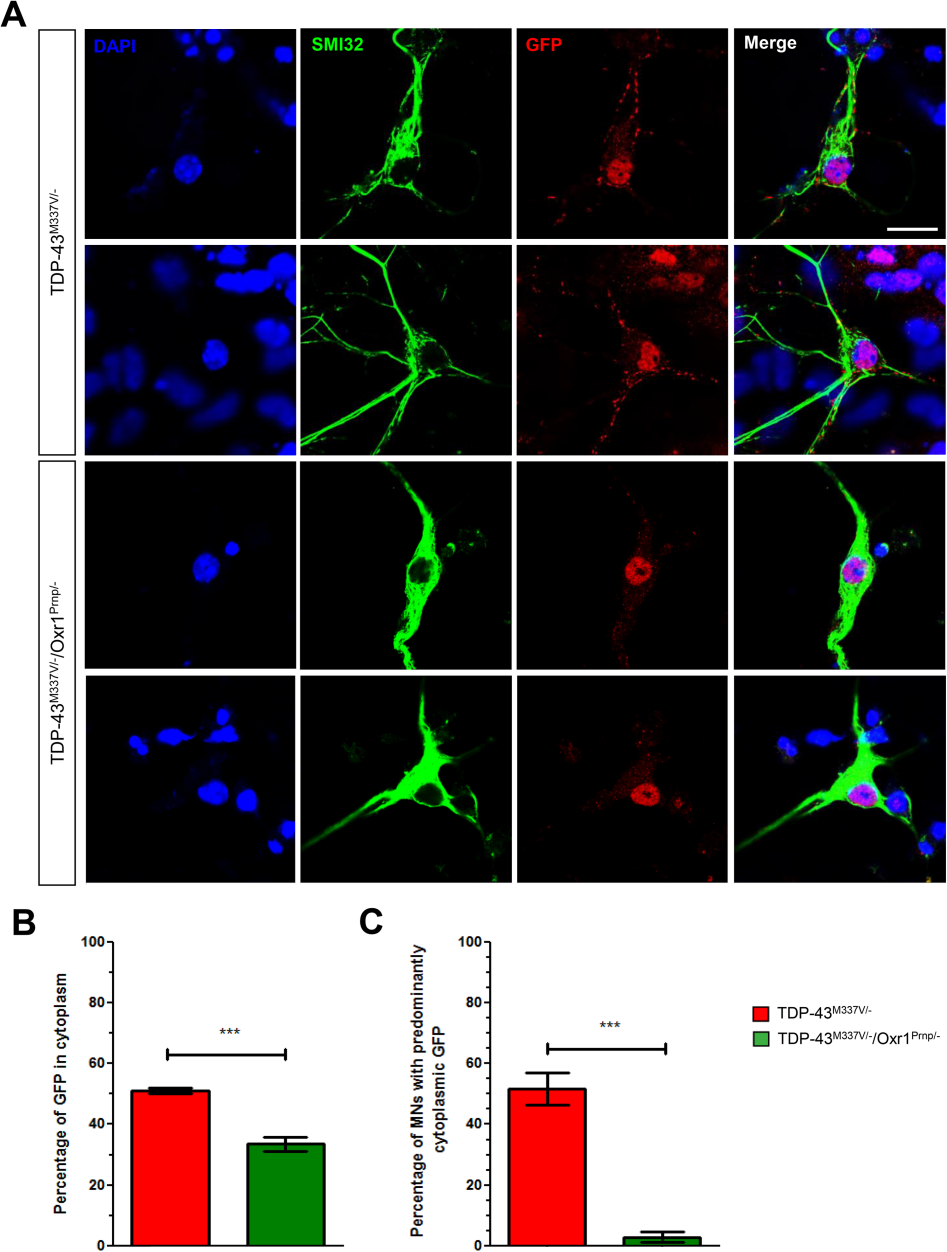
Genetic over-expression of Oxr1 reduces mislocalisation of mutant TDP-43 in TDP-43^M337V/−^ primary motor neurons. (**A**) Representative images of SMI32-positive primary motor neurons from TDP-43^M337V/−^ and TDP-43^M337V/−^/Oxr1^Prnp/−^ mice stained with anti-GFP to quantify Ypet-tagged mutant TDP-43^M337V^. (**B**) Motor neurons from TDP-43^M337V/−^/Oxr1^Prnp/−^ mice display a significant decrease in the proportion of cytoplasmic mutant TDP-43 compared to TDP-43^M337V/−^ mice. (**C**) Motor neurons from TDP-43^M337V/−^/Oxr1^Prnp/−^ animals show a significant decrease in the proportion of motor neurons with >50% cytoplasmic mutant TDP-43 compared with TDP-43^M337V/−^ mice. Values are shown as the mean ± SEM. *N* = 45–60 motor neurons quantified per individual embryonic motor neuron preparation, N = 6 embryos quantified per genotype across two independent litters; ^***^P < 0.001, Unpaired student’s *t*-test. Scale bar: 20 μm.

Our previous cellular data demonstrated that Oxr1 can positively influence the mislocalization of the M337V mutant form of TDP-43 when both are over-expressed exogenously (20). Here, we began by investigating whether this potentially important aspect of Oxr1 function would also be observed in a mammalian genetic system using primary motor neuron cultures where high levels of exogenous expression were not required. For this purpose, we selected the recently described mouse line carrying a single copy of a human TDP-43^M337V^ BAC transgene (33). Primary motor neurons derived from hemizygous (TDP-43^M337V/−^) but not control (TDP-43^WT/−^) embryonic day (E)13.5 embryos have been shown to display robust mislocalization of mutant TDP-43 without exogenous cellular stress, thus providing a novel genetic assay for studying TDP-43 mislocalization and aggregation (33). To investigate whether Oxr1 is a genetic modifier of this key mutant TDP-43-associated phenotype phenotype, homozygous TDP-43^M337V/M337V^ mice were crossed with mice hemizygous for a full-length Oxr1 cDNA transgene driven by a neuronal promoter (Oxr1^Prnp/−^) (21). We chose this breeding strategy such that all the embryos used were experimental, either TDP-43^M337V/−^ or TDP-43^M337V/−^/Oxr1^Prnp/−^ littermates. Of note, the expression of the Oxr1 transgene has already been shown to occur on or before E13.5 (21), and only cells positive for the motor neuron marker SMI32 were used for subsequent analysis (34). After 7 days in culture to reach a sufficient differentiation state, we quantified the localization of the human-specific pool of TDP-43^M337V^ in motor neurons using an antibody directed against its C-terminal Ypet tag (Fig. 1A). Consistent with previous observations (33), a significant proportion of mutant TDP-43 was detected in the cytoplasm of motor neurons from TDP-43^M337V/−^ mice, although interestingly this proportion was significantly reduced in TDP-43^M337V/−^/Oxr1^Prnp/−^ cells (Fig. 1B; *P* = 0.0007). To further investigate this phenotype, the proportion of motor neurons that contained predominantly cytoplasmic mutant TDP-43 was also quantified, revealing a striking 20-fold reduction in cells from TDP-43^M337V/−^/Oxr1^Prnp/−^ animals compared with motor neurons from TDP-43^M337V/−^ mice (Fig. 1C; *P* = 0.0001). Together, these data not only confirm the utility of the TDP-43^M337V^ mouse line for the quantification of mislocalization phenotypes in primary cells, but also reveal that over-expression of Oxr1 reduces the aberrant mislocalization of mutant TDP-43 in TDP-43^M337V/−^ motor neurons, in agreement with our earlier *in vitro* studies (20).

### Over-expression of Oxr1 significantly improves motor function in TDP-43^M337V/M337V^ mice

The data above further support the findings that Oxr1 can improve mutant TDP-43 mislocalisation *in vitro*; however, as we have previously observed the alleviation of neuromuscular dysfunction in the hSOD1^G93A^ mouse model using Oxr1 (21), we wanted to investigate whether this positive modifying effect extended to the mitigation of TDP-43-associated phenotypes *in vivo*. Therefore, given the results from the primary motor neurons above, our next aim was to quantify longitudinal behavioural and neuromuscular phenotypes in the same TDP-43 BAC transgenic model in the presence or absence of Oxr1 over-expression. TDP-43^M337V/−^/Oxr1^Prnp/−^ mice were crossed with TDP-43^M337V/−^ mice to generate experimental cohorts of four genotypic combinations: non-transgenic WTs (NTg), those carrying the Oxr1 transgene alone (Oxr1^Prnp/−^), homozygous TDP-43 mutants (TDP-43^M337V/M337V^) and mice with both transgenes present (TDP-43^M337V/M337V^/Oxr1^Prnp/−^). Using lysates from the spinal cord, we confirmed that that TDP-43^M337V/M337V^ express the Ypet-tagged human TDP-43^M337V^ protein at levels below that of endogenous mouse TDP-43 as previously described (33) (Supplementary Material, Fig. 1A**)**. In addition, we showed that that Oxr1^Prnp/−^ and TDP-43^M337V/M337V^/Oxr1^Prnp/−^ mice express HA-tagged Oxr1 approximately 7-fold higher than endogenous Oxr1 levels, consistent with previous studies of this specific mouse line (Supplementary Material, Fig. 1B; *P* = 0.0003**)** (21). Furthermore, given that Oxr1 has been shown to be induced under a range of stress-associated conditions (17), endogenous Oxr1 protein levels were quantified in the spinal cord, however, the expression was not increased in TDP-43^M337V/M337V^ mutants compared to controls (Supplementary Material, Fig. 1C).

**Figure 2:**
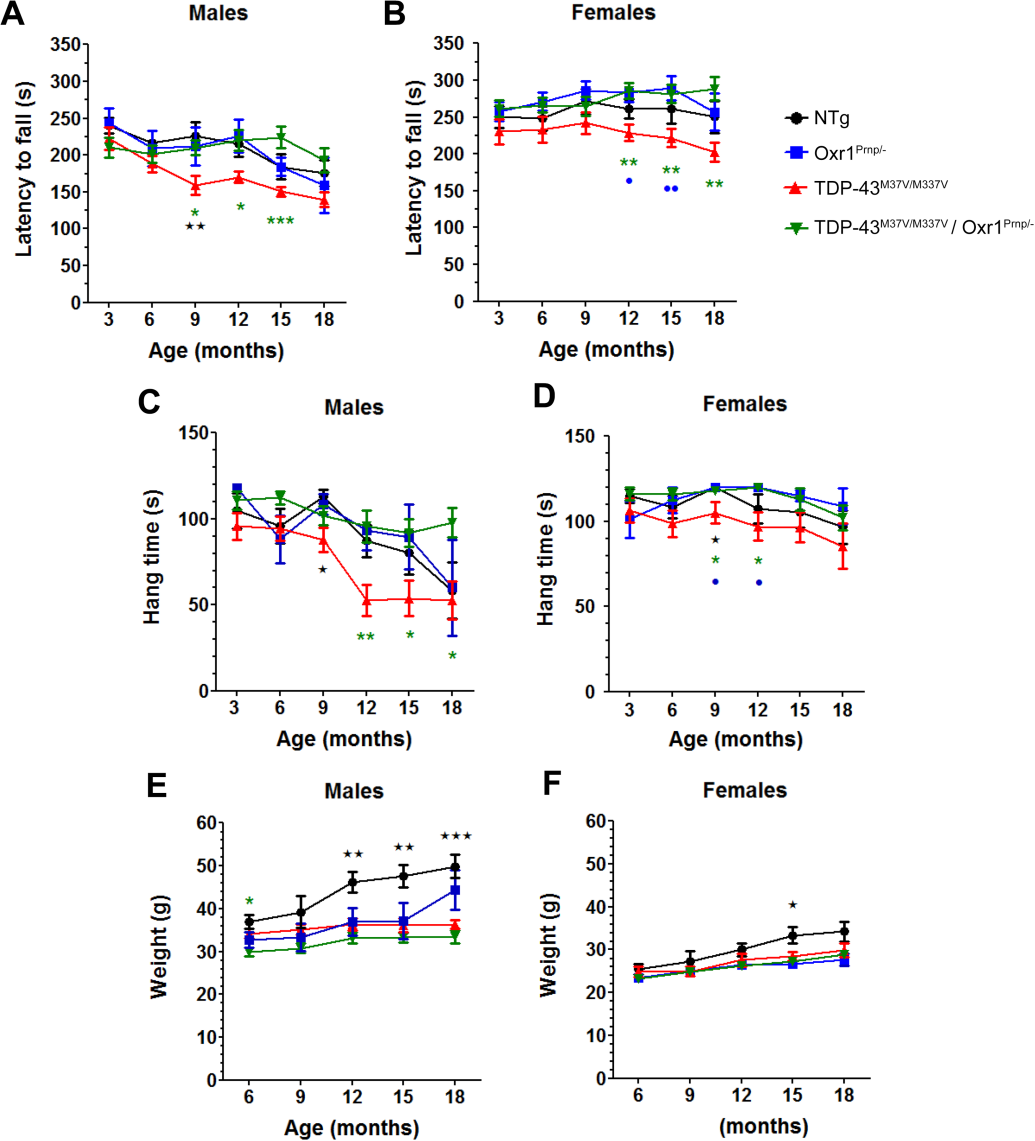
Over-expression of Oxr1 improves motor function and muscle strength in TDP-43^M337V/M337V^ mice. Longitudinal rotarod testing shows a significant improvement in motor coordination for both male (**A**) and female (**B**) TDP-43^M337V/M337V^/Oxr1^Prnp/−^ mice compared to male and female TDP-43^M337V/M337V^ mice, respectively. Grip strength analysis reveals a significant improvement in muscle strength for both male (**C**) and female (**D**) TDP-43^M337V/M337V^/Oxr1^Prnp/−^ mice compared with male and female TDP-43^M337V/M337V^ mice, respectively. TDP-43^M337V/M337V^/Oxr1^Prnp/−^ mice show no significant difference in weight at 9 to 18 months compared to TDP-43^M337V/M337V^ mice for both male (**E**) and female (**F**) cohorts. Values are shown as the mean ± SEM. Sample sizes for each cohort are described in Supplementary Material, Table 1. ^*^P < 0.05, ^**^P < 0.01, ^***^P < 0.001; one-way ANOVA, with Dunnett’s multiple comparison test comparing each group as per the graph legend (green/asterisks, TDP-43^M337V/M337V^/Oxr1^Prnp/−^, blue/circles: Oxr1^Prnp/−^, black/stars: NTg) to TDP-43^M337V/M337V^ mice.

Homozygous TDP-43^M337V/M337V^ mutants have been shown previously to display progressive motor dysfunction from 6 months of age (33); therefore, we used a combination of rotarod and grip strength testing up to 18 months of age to quantify any potential phenotypic modification by Oxr1 over-expression in both males and females (details of the experimental cohorts are shown in Supplementary Material, Table 1). Up to 6 months of age, mice of all four genotypes demonstrated no difference in motor co-ordination or muscle strength (Fig. 2A–D). By 9 months of age, however, male TDP-43^M337V/M337V^ mice begin to show a progressive decrease in motor coordination and muscle strength compared to the control lines as previously described (Fig. 2A and C) (33). This difference in performance did not reach significance compared with every one of the additional genotypes at 9 to 18 months of age, yet importantly, male TDP-43^M337V/M337V^/Oxr1^Prnp/−^ mice performed significantly better than TDP-43^M337V/M337V^ mutants in one or both of the tests at 9, 12, 15 and 18 months of age (Fig. 2A and C; rotarod: *P* = 0.021, *P* = 0.026, *P* = 0.0008, 9–15 months; grip strength: *P* = 0.0071, *P* = 0.028, *P* = 0.037, 12–18 months). Similar to observations in male mice, female TDP-43^M337V/M337V^/Oxr1^Prnp/−^ mice displayed a significant improvement in motor coordination and muscle strength at 12 to 18 and 9 to 12 months of age, respectively, as compared to female TDP-43^M337V/M337V^ littermates (Fig. 2B and D; rotarod: *P* = 0.0084, *P* = 0.0061, *P* = 0.0019, 12–18 months; grip strength: *P* = 0.015, *P* = 0.036, 9–12 months).

As body weight can influence performance in these types of motor-associated tasks (35), we also took weight measurements from the same cohorts of male and female animals (Fig. 2E and F). Critically, these data demonstrated that TDP-43^M337V/M337V^ and TDP-43^M337V/M337V^/Oxr1^Prnp/−^ mice did not show any difference in weight at 9 to 18 months of age within either sex group, suggesting that this was not a factor that could explain the improvement in performance of TDP-43^M337V/M337V^/Oxr1^Prnp/−^ animals versus homozygous TDP-43^M337V^ mutants (Fig. 2A–D). Interestingly, at 12 (*P* = 0.0025), 15 (*P* = 0.0011) and 18 (*P* = 0.0009) months of age, male TDP-43^M337V/M337V^ mutants displayed a significantly decreased body weight compared to NTg WT controls (Fig. 2E); this may explain the lack of statistically significant differences between TDP-43^M337V/M337V^ and NTg mice with regards to motor coordination and muscle strength tests at 12 to 18 months of age, as heavier control mice may be less able to perform the tasks (35). It is noteworthy that, despite a trend towards the NTg females being heavier, there was a significant increase in weight between the female NTg and TDP-43^M337V/M337V^ cohort only at 15 months of age (*P* = 0.041) and overall less of a deterioration of performance in NTg mice (Fig. 2F). Therefore, taken together, these data suggest that over-expression of Oxr1 in neurons is able to improve motor coordination and muscle strength in TDP-43^M337V/M337V^ mice.

### Over-expression of Oxr1 significantly improves muscle denervation and NMJ degeneration in TDP-43^M337V/M337V^ mice

**Figure 3:**
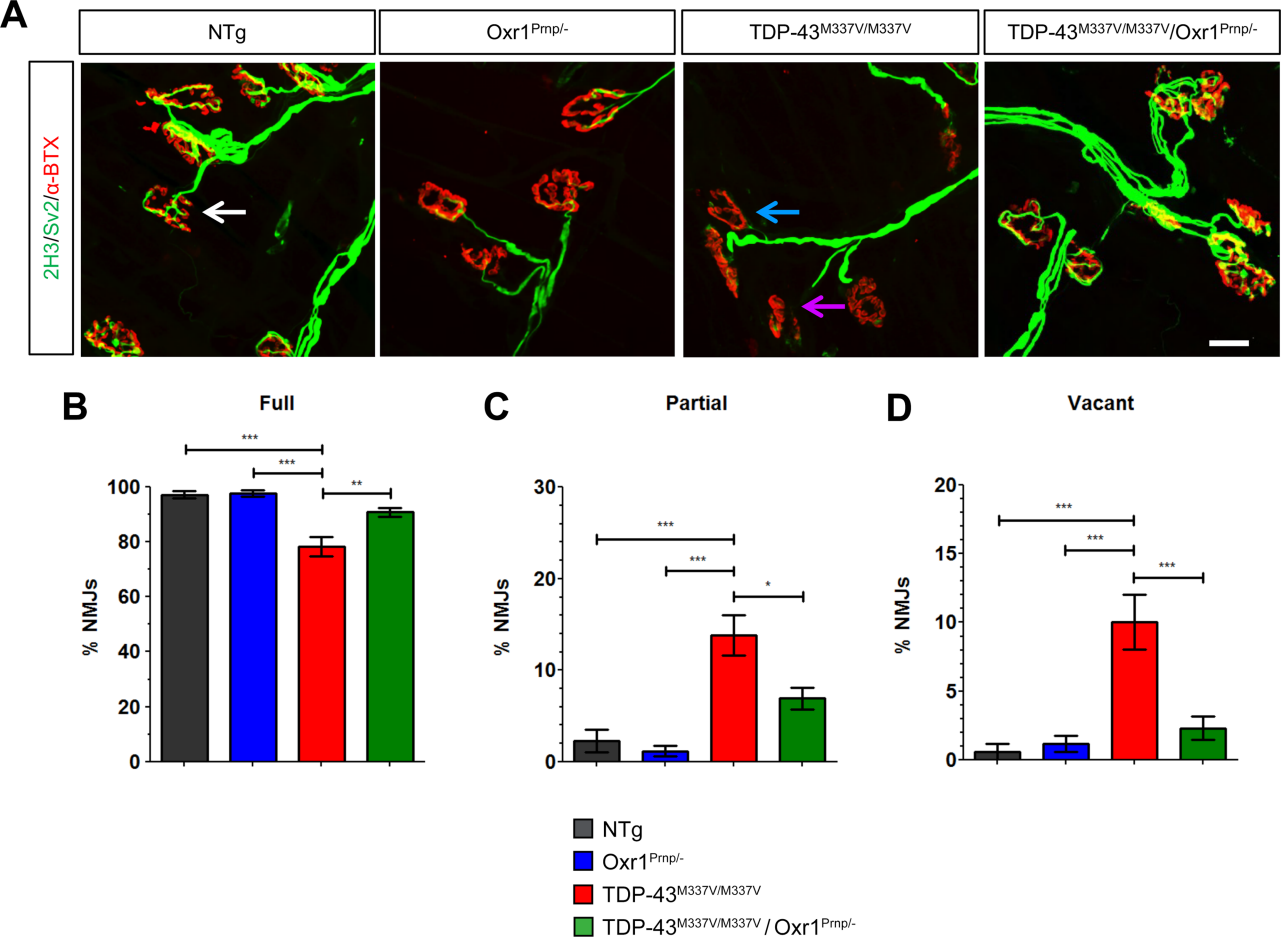
Over-expression of Oxr1 improves neuromuscular innervation in TDP-43^M337V/M337V^ mutants. (**A**) Representative images showing lumbrical NMJs from male NTg, Oxr1^Prnp/−^, TDP-43^M337V/M337V^ and TDP-43^M337V/M337V^/Oxr1^Prnp/−^ mice at 18 months of age. Levels of denervation were assessed by determining the overlap between pre-synaptic (2H3/SV2) and post-synaptic (α-BTX) staining. NMJs were scored as fully innervated (white arrow), partially innervated (blue arrow), or vacant (purple arrow). (**B–D**) Denervation analysis reveals a significant decrease in fully innervated NMJs and a significant increase in partially innervated and vacant NMJs in TDP-43^M337V/M337V^ mice, which is rescued by over-expression of Oxr1. Values are shown as the mean ± SEM. *N* = 3–6 each genotype, *N* > 58 NMJs per mouse. ^*^P < 0.05, ^**^P < 0.01, ^***^P < 0.001; one-way ANOVA with Dunnett’s multiple comparison test comparing each group to TDP-43^M337V/M337V^ mice. Scale bar: 20 μm.

Muscle denervation and degeneration of the NMJs is a major defining feature of ALS, and may occur prior to lower motor neuron loss (36–38). Furthermore, it has been demonstrated that rodent models over-expressing mutant TDP-43^M337V^ display muscle denervation and abnormal NMJ morphology (39,40). In the TDP-43^M337V/M337V^ mutant studied here, a small but significant increase in the number of partially denervated NMJs was reported at 12 months of age in hind-paw lumbrical muscles (33). Therefore, we next examined whether Oxr1 over-expression was able to recover or delay this specific disease-relevant phenotype, and importantly, at a more advanced symptomatic time-point than had been previously studied in this line. Hind-paw lumbrical muscles from 18-month-old male mice were stained with antibodies for presynaptic nerve terminals (SV2 and 2H3) and post-synaptic acetylcholine receptors located on the motor endplate (α-BTX). The number of NMJs that were fully innervated, partially innervated or fully denervated were then quantified (Fig. 3A–D). We observed that TDP-43^M337V/M337V^ mice show a significant decrease in the number of fully innervated NMJs at 18 months of age compared with both the NTg and Oxr1^Prnp/−^ controls (Fig. 3B; *P* < 0.001 versus both control lines). In addition, TDP-43^M337V/M337V^ mice displayed an approximately 10-fold increase in the number of partially innervated NMJs and vacant NMJs compared with the same control lines (Fig. 3C and D; *P* < 0.0001 versus both control lines). Importantly, we also observed a significant increase in the number of fully innervated NMJs in TDP-43^M337V/M337V^/Oxr1^Prnp/−^ mice versus TDP-43^M337V/M337V^ littermates (Fig. 3B; *P* = 0.0082). Consistent with these data, we found a significant 2- to 4-fold decrease in the number of partially innervated (*P* = 0.025) and vacant (*P* = 0.0008) NMJs compared to TDP-43^M337V/M337V^ mutants (Fig. 3C and D). Together, these data not only reveal a robust lumbrical muscle denervation phenotype in TDP-43^M337V/M337V^ mice at 18 months of age, but also that this key disease-relevant phenotype can be significantly rescued by over-expression of Oxr1.

**Figure 4:**
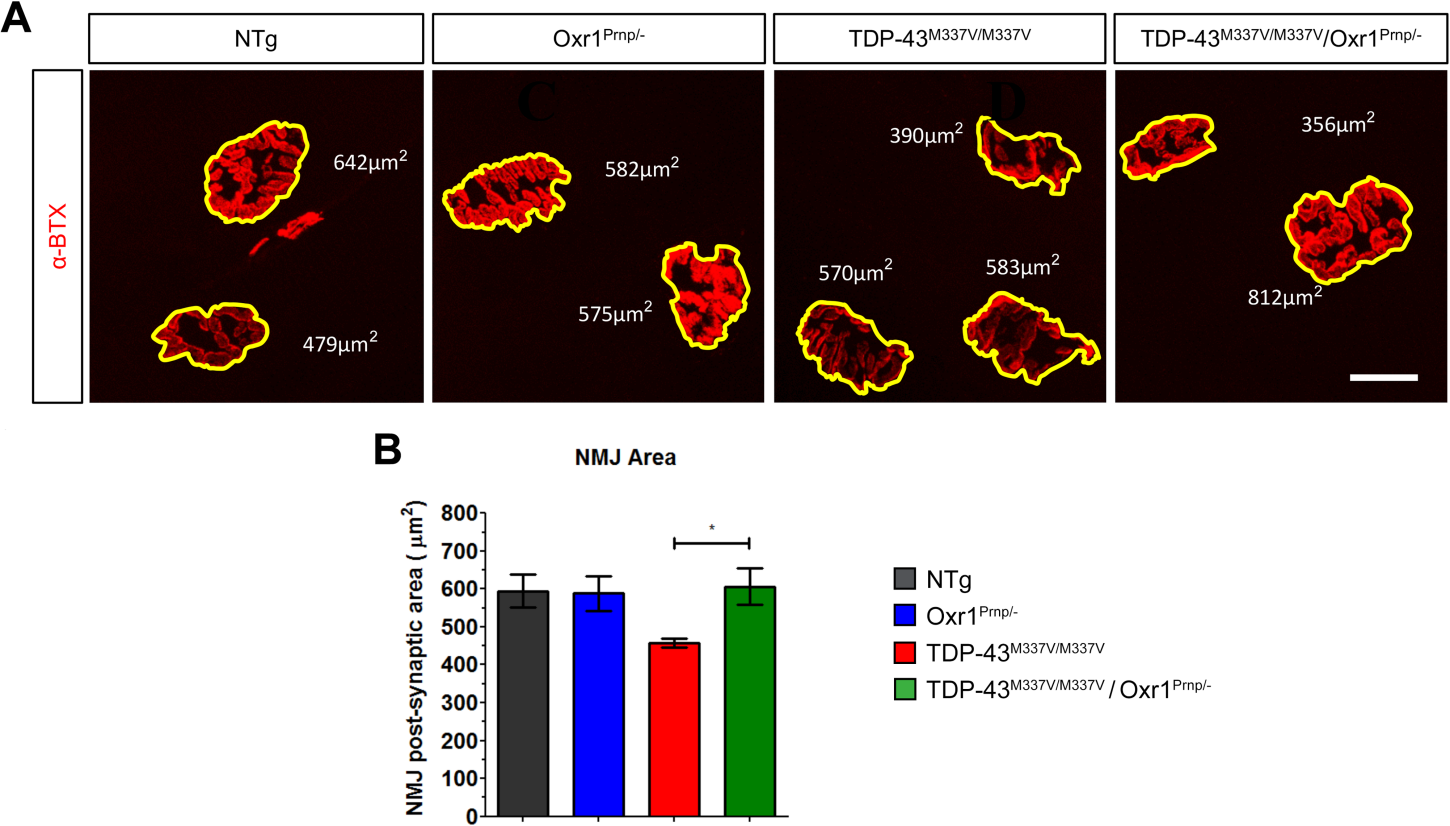
Over-expression of Oxr1 improves post-synaptic NMJ integrity in TDP-43^M337V/M337V^ mutants. (**A**) Representative images of motor endplates used to measure post-synaptic area from male NTg, Oxr1^Prnp/−^, TDP-43^M337V/M337V^ and TDP-43^M337V/M337V^/Oxr1^Prnp/−^ mice at 18 months of age. (**B**) Average motor endplate area is reduced in TDP-43^M337V/M337V^ mice, and is rescued by over-expression of Oxr1. Values are shown as the mean ± SEM. *N* = 3–6 each genotype, *N* = 30–50 NMJs per mouse. ^*^P < 0.05; one-way ANOVA with Dunnett’s multiple comparison test comparing each group to TDP-43^M337V/M337V^ mice. Scale bar: 20 μm.

During the progression of ALS, muscle fibers degenerate and regenerate, which causes alterations in the size of post-synaptic motor endplates (41); hence, immunostaining of these structures can be used as a secondary indicator of muscle denervation. Therefore, we went on to investigate the area of post-synaptic endplates from lumbrical muscle NMJs in our experimental cohorts at 18 months of age (Fig. 4A). The average end-plate area was ~25% lower in male TDP-43^M337V/M337V^ mice compared to NTg and Oxr1^Prnp/−^ controls, consistent with endplate degeneration in homozygous mutants, although this did not reach significance (Fig. 4B; *P* = 0.067, *P* = 0.071 versus NTg and Oxr1^Prnp/−^, respectively). Importantly, however, TDP-43^M337V/M337V^/Oxr1^Prnp/−^ mice displayed a very similar average post-synaptic endplate area compared to both NTg and Oxr1^Prnp/−^ animals, with a significant increase compared to TDP-43^M337V/M337V^ mutants (*P* = 0.031), suggesting that motor endplate degeneration in TDP-43^M337V/M337V^ mutants can be rescued by over-expression of Oxr1 (Fig. 4B).

To confirm that these pathological data are consistent in females, we carried out an equivalent set of NMJ analyses from 18-month-old female TDP-43^M337V/M337V^ and TDP-43^M337V/M337V^/Oxr1^Prnp/−^ littermates. Similar to the data observed in males, female TDP-43^M337V/M337V^/Oxr1^Prnp/−^ mice display a significant increase in fully innervated NMJs (*P* = 0.044) and significant decrease in vacant NMJs (*P* = 0.0077) compared to TDP-43^M337V/M337V^ littermates (Supplementary Material, Fig. 2A–D). In addition, the average post-synaptic motor endplate area was significantly greater in female TDP-43^M337V/M337V^/Oxr1^Prnp/−^ mice compared with female TDP-43^M337V/M337V^ mutants (Supplementary Material, Fig. 2E and F; *P* = 0.019). Together, these data indicate that over-expression of Oxr1 significantly improves lumbrical muscle denervation and NMJ degeneration in TDP-43^M337V/M337V^ mice at 18 months of age.

Previous neuropathological analysis of TDP-43^M337V/M337V^ mutants reported no detectable mislocalization of either endogenous or human mutant TDP-43 up to 24 months of age and no loss of motor neurons in the spinal cord at 12 months (33). We independently quantified both of these parameters at 18 months of age in our experimental cohort, and in agreement, found no significant decrease in the number of lower motor neurons in the spinal cord of TDP-43^M337V/M377V^ mice or an increase in the number of motor neurons with detectable cytoplasmic TDP-43 aggregates compared to controls (Supplementary Material, Fig. 3A and B). These data confirm that the neuromuscular defects we observed in TDP-43^M337V/M337V^ mice occur in the absence of detectable TDP-43 aggregation in lower motor neurons.

### Over-expression of Oxr1 improves muscle pathology observed in TDP-43^M337V/M337V^ mutants

**Figure 5:**
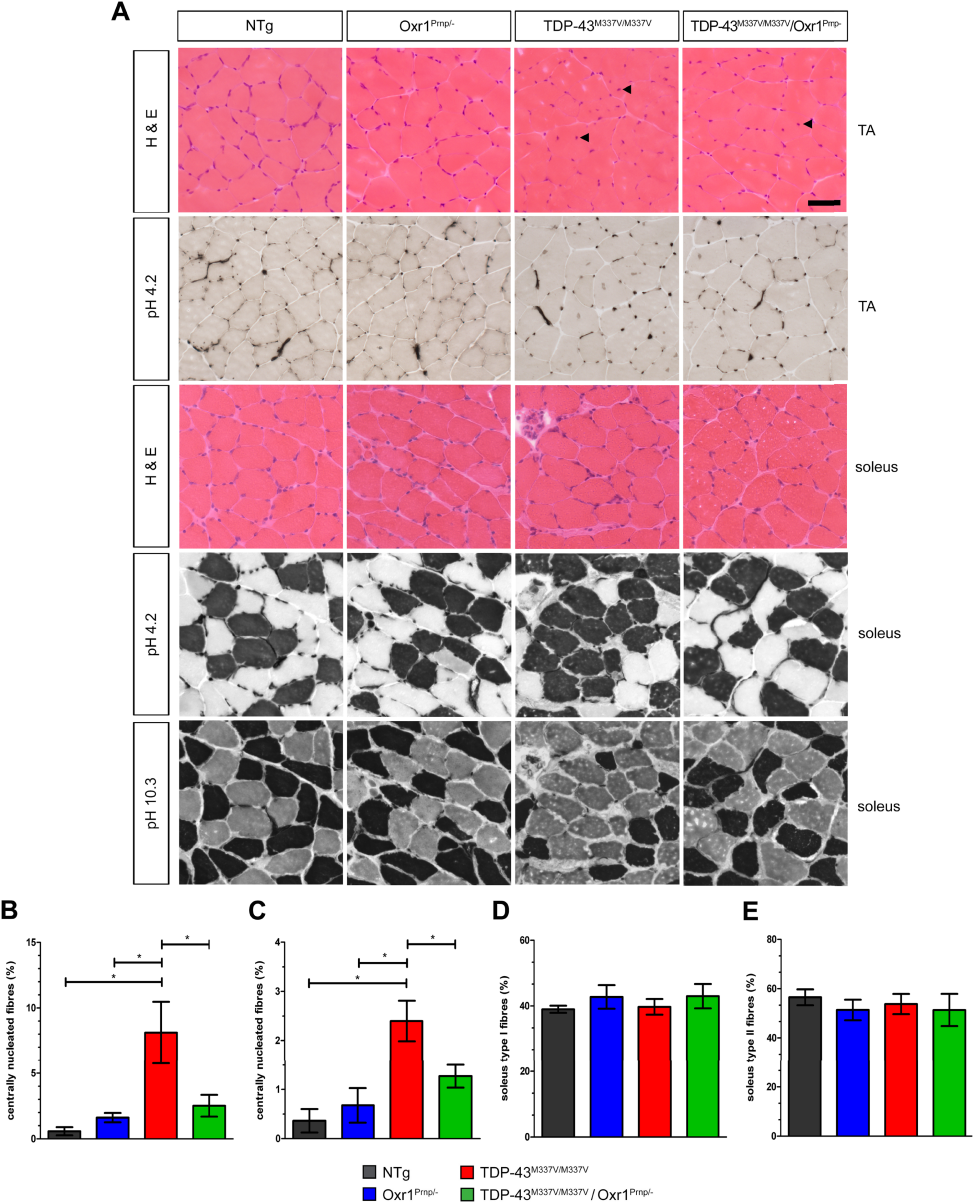
Over-expression of Oxr1 improves muscle pathology in male TDP-43^M337V/M337V^ mice. (**A**) Representative images of H&E-stained TA and soleus muscle fibre cross-sections from NTg, Oxr1^Prnp/−^, TDP-43^M337V/M337V^ and TDP-43^M337V/M337V^/Oxr1^Prnp/−^ mice at 18 months of age. Examples of centrally nucleated fibres are indicated (arrowheads). Representative images of ATPase staining for muscle fibre types using the preincubation pH conditions as indicated are also shown; pH 4.2, type I fibres dark staining and type II fibres light staining; pH 10.3, type I fibres light staining and type II fibres dark staining. (**B** and **C**) The percentage of centrally nucleated fibres per section is significantly increased in TDP-43^M337V/M337V^ mice in both TA (B) and soleus (C), which is rescued by over-expression of Oxr1. (**D** and **E**) Quantitation of fibre-type staining in the soleus reveals no significant differences in the proportions between genotypes, although fibre-type grouping is observed in TDP-43^M337V/M337V^ mice (A). Values are shown as the mean ± SEM. *N* = 3–5 male animals per genotype. ^*^P < 0.05, One-way ANOVA with Dunnett’s multiple comparison test comparing each group to TDP-43^M337V/M337V^ mice. Scale bar: 100 μm.

Muscle atrophy is another hallmark of ALS pathology that occurs concomitantly with muscle denervation and NMJ degeneration (42–44). Therefore, given the muscle denervation we observed in TDP-43^M337V/M337V^ mutants, we next determined whether detectable muscle pathology occurs at the same timepoint, as this has not been reported previously in this line. In ALS, muscles that are largely composed of fast-twitch fibres appear to be more vulnerable to degeneration prior to motor neuron loss (43,44). Thus we chose to begin by examining tibialis anterior (TA) muscles as these contain a high proportion of fast-twitch fibres compared to the gastrocnemius and soleus muscles in C57BL6/J mice (45). Initially, we observed no overt signs of muscle atrophy, such as decreased fibre number, in TA muscle from TDP-43^M337V/M337V^ mutants or any of the control strains (Fig. 5A). However, quantification of centrally-nucleated fibres, a marker of muscle fibre degeneration and subsequent regeneration or myopathy revealed a significant increase in TDP-43^M337V/M337V^ mice compared to NTg and Oxr1^Prnp/−^ controls at 18 months of age (Fig. 5B; *P* = 0.011, *P* = 0.026 versus NTg and Oxr1^Prnp/−^, respectively). Moreover, and in-line with our NMJ data, the number of centrally nucleated fibres was significantly lower in TDP-43^M337V/M337V^/Oxr1^Prnp/−^ mice compared to TDP-43^M337V/M337V^ mutants (Fig. 5B, *P* = 0.039), suggesting that over-expression of Oxr1 improves hind limb muscle pathology in the TDP-43^M337V/M337V^ line. Next, we carried out the same analysis on the soleus muscle from all four genotypes (Fig. 5A). Although a smaller percentage of centrally nucleated fibres were detected overall compared to the TA, there was a significant increase in TDP-43^M337V/M337V^ mice compared with controls, and significantly fewer in TDP-43^M337V/M337V^/Oxr1^Prnp/−^ mice compared with TDP-43^M337V/M337V^ mutants (Fig. 5C; *P* = 0.020, *P* = 0.033, *P* = 0.041 versus NTg, Oxr1^Prnp/−^ and TDP-43^M337V/M337V^/Oxr1^Prnp/−^, respectively). To investigate this further, we went on to determine whether there was evidence for muscle fibre-type alterations in TDP-43^M337V/M337V^ animals, such grouping or switching to different fibre-types as observed in human ALS and neurogenic myopathies due to muscle remodelling (46). Quantitative ATPase histological staining was carried out to delineate between slow (type I) and fast (type II) fibre types based-on histological pH-driven inhibition of myosin-ATPase enzymes. Despite evidence for muscle fibre myopathy and/or regeneration in TA muscle from TDP-43^M337V/M337V^ mice, virtually no type I muscles were observed in any of the four genotypes as expected in normal, aged samples (47) (Fig. 5A). Therefore, next we carried out fibre-type analysis on the soleus muscle of the same mice as this muscle group consists of a larger proportion of type I fibres (45). Quantification using a pH 4.2 pre-incubation buffer to stain type I fibres, alongside a pH 10.3 buffer to stain type II fibres in a reciprocal manner, showed there was no significant change in the overall proportion of fibre types between the genotypes (Fig. 5D and E); interestingly, however, examples of fibre-type grouping were only observed in TDP-43^M337V/M337V^ samples (Fig. 5A), suggesting that muscle remodelling due to denervation and reinnervation occurs in TDP-43^M337V/M337V^ mice that can be improved by Oxr1 over-expression.

### Neuroinflammation occurs in the spinal cord of TDP-43^M337V/M337V^ mice and is alleviated by Oxr1 over-expression

**Figure 6:**
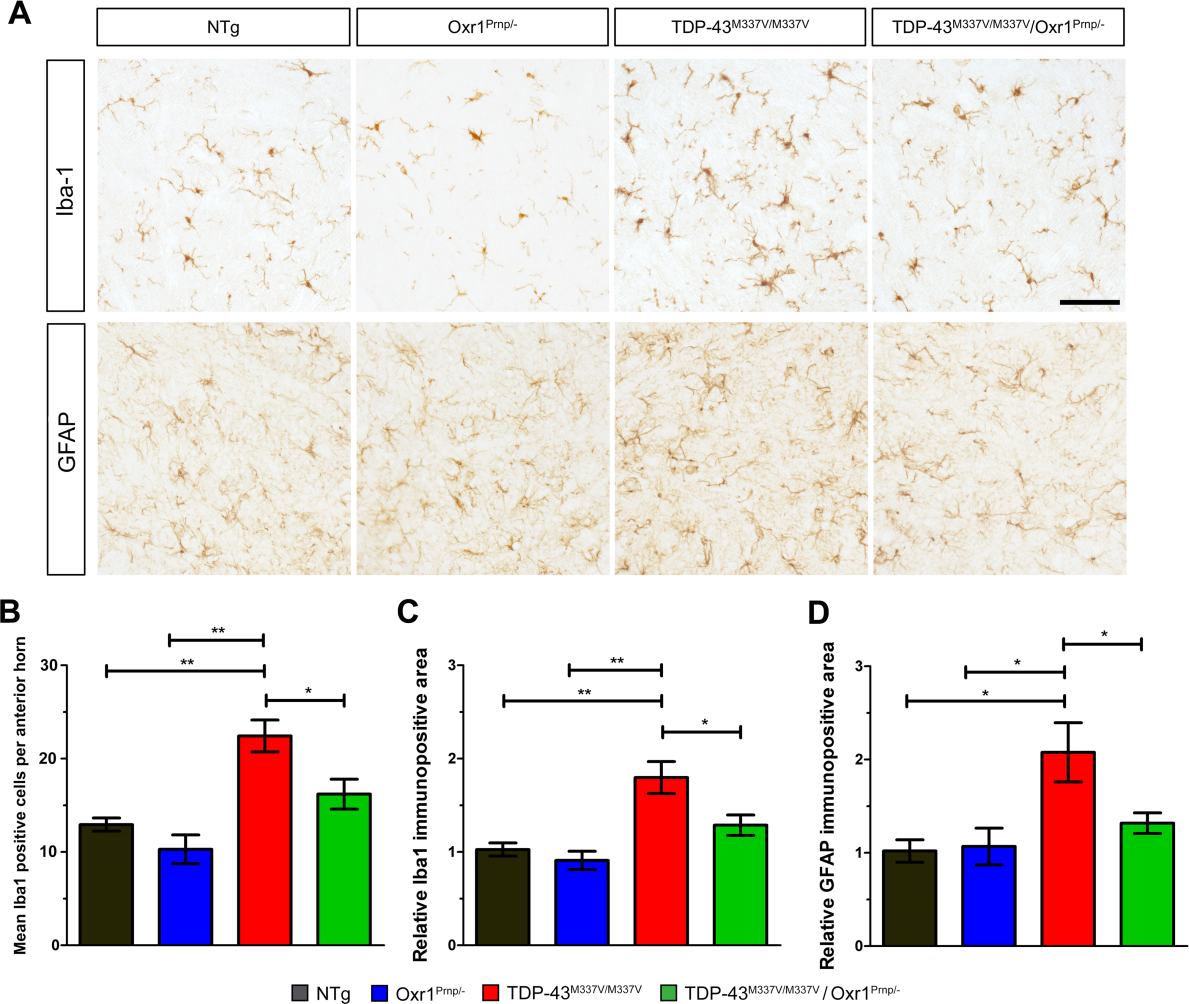
Neuroinflammation in lumbar spinal cords from male TDP-43^M337V/M337V^ mice is alleviated by Oxr1 over-expression. (**A**) Representative images of spinal cord sections stained with anti-Iba1 and anti-GFAP from male NTg, Oxr1^Prnp/−^, TDP-43^M337V/M337V^ and TDP-43^M337V/M337V^/Oxr1^Prnp/−^ mice. (**B** and **C**) Quantification of the number of immunopositive cells and immunopositive area for anti-Iba1 reveals increased microgliosis in TDP-43^M337V/M337V^ mice that is significantly reduced in TDP-43^M337V/M337V^/Oxr1^Prnp/−^ animals. (**D**) Quantification of the immunopositive area for anti-GFAP signal reveals increased astrogliosis in TDP-43^M337V/M337V^ mice that is significantly reduced in TDP-43^M337V/M337V^/Oxr1^Prnp/−^ animals. (B–D) Values are shown as the mean ± SEM. N = 3–5 animals per genotype; ^*^P < 0.05, ^**^P < 0.01; One-way ANOVA with Dunnett’s multiple comparison test comparing each group to TDP-43^M337V/M337V^ mice. Scale bar: 100 μm.

Neuroinflammation is predicted to play an active role during disease progression in ALS patients, and has been observed in mouse models over-expressing ALS-associated proteins including M337V mutant TDP-43 (31,48–50). In the TDP-43^M337V/M337V^ BAC transgenic line at 12 months of age, ionized calcium binding adaptor molecule 1 (Iba1) immunostaining indicated no overt microgliosis in the spinal cord (33). However, we wanted to assess whether either astrogliosis or microgliosis could be observed at the same later symptomatic timepoint as our other pathological observations, but also not be confounded by motor neuron death. Therefore, lumbar spinal cord sections were immunostained for Iba1 and glial fibrillary acidic protein (GFAP) from TDP-43^M337V/M337V^ mice and NTg controls at 18 months of age. We observed that the ventral horns of TDP-43^M337V/M337V^ mice display significantly increased immunoreactivity for Iba1, both in terms of the number of immunopositive cells (Fig. 6A and B; *P* = 0.0064, *P* = 0.0088 versus NTg and Oxr1^Prnp/−^, respectively) and immunopositive staining area (Fig. 6A and C; *P* = 0.0075, *P* = 0.0073 versus NTg and Oxr1^Prnp/−^, respectively). For GFAP, increased immunoreactivity was also observed compared to the control lines (Fig. 6A and D; *P* = 0.033, *P* = 0.041 versus NTg and Oxr1^Prnp/−^, respectively), indicative of an increased neuroinflammatory response in mutant animals.

It has been demonstrated previously that over-expression of Oxr1 in neurons of hSOD1^G93A^ mice decreased astrogliosis and microgliosis in the spinal cord, suggesting that Oxr1 is able to influence neuroinflammation *in vivo* (21). Therefore, in parallel, we determined whether neuronal over-expression of Oxr1 could ameliorate the neuroinflammation observed in TDP-43^M337V/M337V^ mice. Interestingly, we observed a significant 25% decrease in the number of Iba1 immunopositive cells (Fig. 6A and B; *P* = 0.016) and immunoreactive area (Fig. 6A and C; *P* = 0.028) from TDP-43^M337V/M337V^/Oxr1^Prnp/−^ spinal cord sections compared to homozygous mutants. The GFAP immunoreactive staining area was also significantly decreased in TDP-43^M337V/M337V^/Oxr1^Prnp/−^ compared to TDP-43^M337V/M337V^ littermates (Fig. 6A and D; *P* = 0.024 versus TDP-43^M337V/M337V^/Oxr1^Prnp/−^). Taken together, these data indicate that an induction of the inflammatory response occurs in the TDP-43^M337V/M337V^ mutant spinal cord, with neuronal Oxr1 over-expression facilitating a dampening of the observed microgliosis and astrogliosis.

### Oxr1 binds to TDP-43 *in vivo*

**Figure 7:**
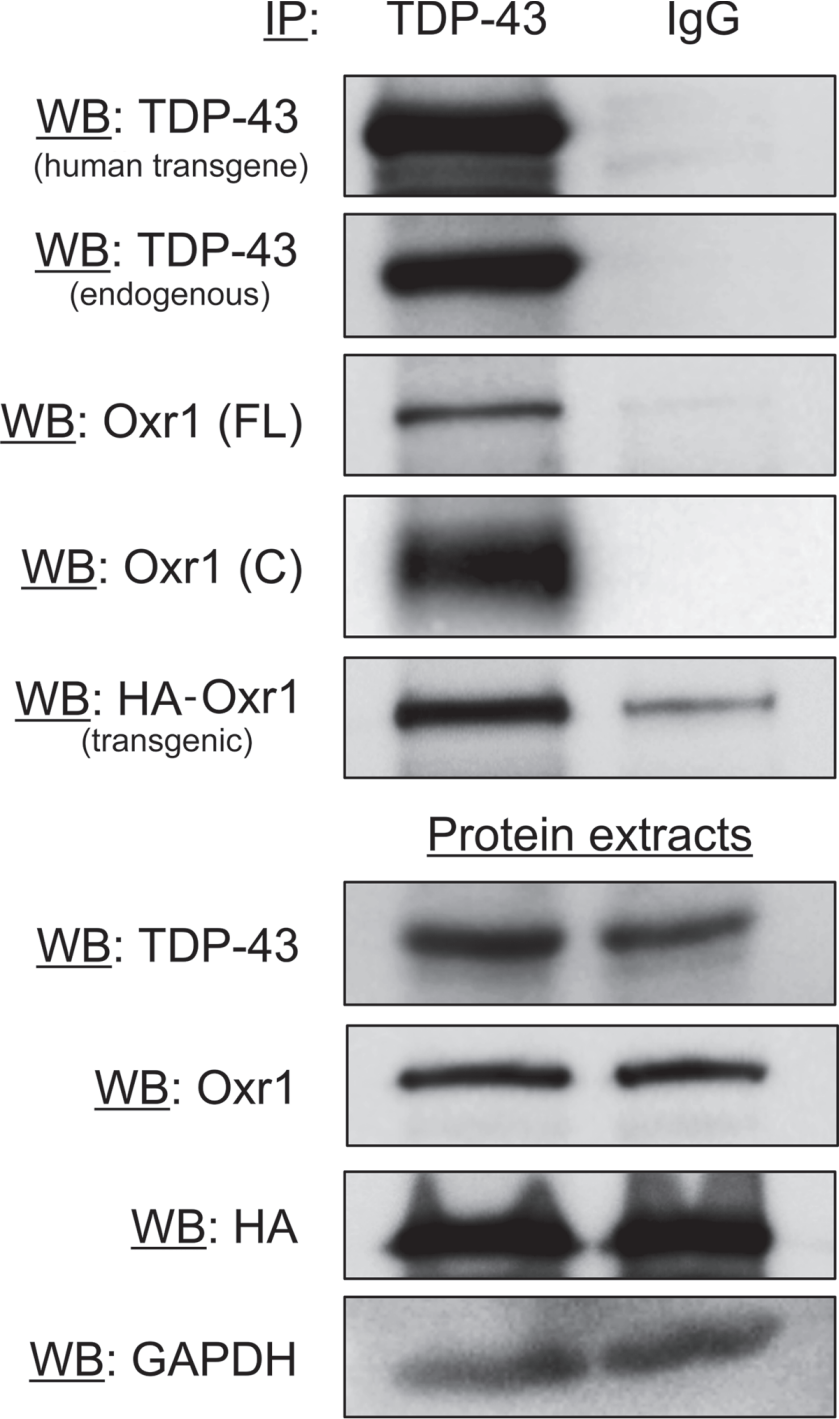
Oxr1 binds to TDP-43 *in vivo*. Co-IP in spinal cord tissue from a TDP-43^M337V/M337V^/Oxr1^Prnp/−^ mouse using a pan-TDP-43 antibody (left lanes) and non-specific IgG (right lanes). Lower panels represent input loading controls. Antibodies used for western blotting are indicated. The Oxr1 antibody recognises both the TLDc domain-containing endogenous full-length Oxr1-(FL) and the shortest isoform Oxr1-(C).

We have demonstrated previously that over-expression of Oxr1 *in vitro* alongside exogenous M337V mutant TDP-43 can improve TDP-43 mislocalization under oxidative stress, and this phenomenon may rely on an interaction between the two proteins (20). Thus, our new data in primary motor neurons from TDP-43^M337V/−^/Oxr1^Prnp/−^ mice (Fig. 1) is important, as it further supports a role for Oxr1 in preventing TDP-43 mislocalisation in a mammalian genetic system. Therefore, to determine whether binding between TDP-43 and Oxr1 occurs *in vivo*, co-immunoprecipitation (IP) experiments were carried out from spinal cord tissue. Using a pan-TDP-43 antibody, both the full-length Oxr1 isoform (Oxr1-FL) and the shorter Oxr1-C isoform were co-immunoprecipitated from TDP-43^M337V/M337V^/Oxr1^Prnp/−^ spinal cord (Fig. 7). Next, in order to detect an interaction between TDP-43 and the HA-tagged Oxr1 transgene, we also immunoblotted with an anti-HA antibody. HA-tagged Oxr1 successfully co-immunoprecipitated with TDP-43 with a much higher efficiency than the control (IgG only) (Fig. 7). Together, these data demonstrate that TDP-43 can bind to two functionally important endogenous Oxr1 isoforms *in vivo,* as well as the Oxr1 transgene overexpressed in TDP-43^M337V/M337V^/Oxr1^Prnp/−^ mice.

## Discussion

The central role for TDP-43 in ALS and FTD pathophysiology, alongside the discovery of causative mutations in fALS, has prompted the generation of multiple rodent TDP-43 disease models. Yet the broad phenotypic variation between mutant lines likely reflects non-specific toxicity of TDP-43 transgenes driven by artificial promoters (24,51). Consequently, there has been an effort to generate models that express the protein at more physiologically-relevant levels using endogenous promoters. Here, we have studied one such novel transgenic line containing the entire human TDP-43 locus carrying a pathogenic M337V mutation. Experimental animals thus express the mutant human protein in addition to endogenous Tdp-43, a situation akin to the dominant nature of M337V mutations observed in ALS (52). Our own additional pathological studies of these TDP-43^M337V/M337V^ mice revealed significant denervation at the NMJ at a later timepoint than had been studied previously (33), in addition to newly discovered hind-limb muscle pathology and neuroinflammation in the lumbar spinal cord. These findings further demonstrate the utility of this mouse line for the study of disease-relevant pre-symptomatic interventions in a model where the neuromuscular system has developed normally prior to neurodegeneration, as occurs in human ALS.

We went on to show that the neuromuscular defects we observed in TDP-43^M337V/M337V^ mice at 18 months of age occur in the absence of detectable TDP-43 aggregation in lower motor neurons. These data are in agreement with a number of other *in vivo* models that have analysed the same pathogenic mutation. For example, induction of a motor neuron-specific TDP-43^M337V^ transgene in neonatal rats results in motor dysfunction, rapid motor neuron loss, NMJ degeneration and premature death by 3 months of age, yet without the presence of TDP-43-positive aggregates in lower motor neurons (39). Similarly, driving hTDP-43^M337V^ expression using a neuronal Thy1.2 promoter results in dose-dependent neuromuscular defects and early mortality in mice; however, TDP-43 mislocalization was not associated with these particular phenotypes (53). Most recently, a mouse knock-in model has been described in which the M337V mutation was introduced into the endogenous mouse Tdp-43 locus (48). Interestingly, the phenotype of mutants carrying either one or two copies of this particular mutant knock-in allele is somewhat subtler than the human BAC model we have utilized here. For example, homozygous M337V knock-in mice displayed a small but significantly reduced innervation of TA muscles, although this was only detectable at 30 months of age. In addition, an increase in gliosis was described in the lumbar spinal cord at 24 months in homozygous M337V knock-in mice compared to controls, although no mislocalization of TDP-43 was detected (48). Hence these findings add to the growing body of evidence suggesting TDP-43 aggregation is not primarily responsible for the onset of disease symptoms in ALS. Although we cannot rule-out the presence of mislocalized TDP-43 species that are beyond antibody detection levels in TDP-43^M337V/M337V^ mice, neither neurofilament, peripherin nor p62-containing aggregates were reported previously in this BAC transgenic model up to 24 months of age (33). It is noteworthy that the mouse endogenous knock-in M337V allele rescued the lethality of a *Tdp-43* knockout mutant, suggesting this mutation is not completely a loss-of-function mutation (48). In contrast, a similar experiment revealed that the human TDP-43^M337V^ BAC transgene did not rescue mice lacking endogenous Tdp-43 (33); this difference is likely largely due to the lower relative expression level of the BAC-derived mutant TDP-43 compared with endogenous protein (Supplementary Material, Fig. 1A), although different functional targets of the human and mouse protein that are critical for development may also play a role.

Previously we successfully utilized over-expression of Oxr1 to delay neuromuscular abnormalities in the hSOD^G93A^ mouse model (21). Here, using the same genetic approach to over-express Oxr1 in neurons, we demonstrate a striking reduction in lumbrical muscle denervation and NMJ degeneration in TDP-43^M337V/M337V^/Oxr1^Prnp/−^ mice versus homozygous TDP-43^M337V^ mutants. Considering that lumbrical muscles in the paws are directly involved in grip strength and locomotion, the protection Oxr1 provides against NMJ degeneration and muscle denervation likely explains why we also observe significantly improved motor function in TDP-43^M337V/M337V^/Oxr1^Prnp/−^ mice compared with TDP-43^M337V/M337V^ littermates. Although the molecular function of OXR1 is still under investigation, there is growing evidence that this protein can protect against oxidative stress via the modulation of antioxidants and related cell death-associated pathways (17,54–56). Indeed, there is evidence that presynaptic nerve terminals at the NMJ are sensitive to such reactive oxygen species, as under oxidative stress conditions neurotransmitter release machinery can be compromised, thus potentiating neurodegeneration (57).

An additional hypothesis associated with Oxr1’s neuroprotective properties *in vivo* relates to the marked delay in neuroinflammation observed in the spinal cord of hSOD1^G93A^ mutants expressing exogenous Oxr1 (21). One possibility linking Oxr1 to inflammation would be that Oxr1 affects the function of proteins participating in the immune response. For example, we recently demonstrated that Oxr1 modulates the activity of glucose-6-phosphate isomerase (Gpi1) and peroxiredoxin 2 (Prdx2), which can be secreted by neurons to activate glial cells and thus contribute to the inflammatory response. (55,58–60). Another possibility would be that Oxr1 over-expression leads to a global reduction of ROS levels within neurons, leading to a reduction in proinflammatory signals released by neurons, which is often associated with sustained oxidative stress conditions and an inhibition of inflammation in the central nervous system (61). In the TDP-43^M337V/M337V^ model we observed an increase in activated microglia and astrocytes at 18 months of age in the spinal cord. We provide evidence, based on immunohistochemistry, that the activation of microglia and astrocytes was significantly reduced by the presence of exogenous Oxr1, although this was not completely prevented by neuronal Oxr1 over-expression. This discrepancy could be explained by a potential specificity of action of Oxr1 on sub-populations of activated glial cells. Indeed, both the phenotype and function of both microglia and astrocytes evolve during the immune response from cut neuroprotective to a pro-inflammatory cut phenotype (62,63). Therefore, further studies would be required to characterize the ‘reactive profile’ of glial cells in hTDP-43^M337V^ mice and to determine the precise influence of Oxr1 on microglia and astrocytes during inflammation. Nevertheless, we have shown here that the positive effects of Oxr1 over-expression are not specific to mutant SOD1-associated phenotypes but also beneficial to other forms of ALS, further supporting the efficacy of Oxr1 as a valuable modifier of neuromuscular dysfunction *in vivo*.

An additional feature of this hTDP-43^M337V^ model is the presence of a robust TDP-43 mislocalisation phenotype in cultured motor neurons (16,33). Why TDP-43 shows a propensity to aggregate in culture but not in the spinal cord of hTDP-43^M337V^ mice remains unclear, although this in-part likely reflects the lack of supporting cells *in vitro* that are present *in vivo* (64). We were able to take advantage of this valuable experimental system and demonstrate that genetically-driven over-expression of Oxr1 leads to a significant reduction in mislocalized mutant TDP-43 in motor neurons. Furthermore, we demonstrated an interaction between TDP-43 and endogenous Oxr1 isoforms in addition to transgenic over-expressed Oxr1 in the spinal cord. These findings extend our former data in N2a cells, where TDP-43 mutants that were able to bind to Oxr1, including TDP-43^M337V^, showed a tendency to relocate to the nucleus when Oxr1 was over-expressed (20). Therefore, our hypothesis is that an interaction with Oxr1 either disrupts aggregate formation or promotes the correct cellular transportation of TDP-43. The importance of the nucleocytoplasmic transport machinery to this key aspect of TDP-43 biology is becoming increasingly apparent (65) thus it is interesting that our earlier Oxr1 interaction studies identified components of this particular system under oxidative stress (20). For example, RAN (a member of the RAS oncogene family), one such putative Oxr1 binding partner, is a small GTPase and an essential regulator of nuclear import. Specifically, it has been established that RAN is required for the normal nuclear localization of TDP-43 (66) in concert with a feedback loop whereby TDP-43 can regulate the expression of RAN itself (67). As rescuing TDP-43 mislocalisation may be a critical approach for tackling TDP-43 proteinopathies, the direct involvement of Oxr1 in these trafficking processes warrants further investigation in the future.

With the availability of rodent models of TDP-43-associated ALS, one valuable experimental strategy is to find modifiers of disease using a genetic approach (68,69). Here we demonstrate that Oxr1 is the first antioxidant protein that, when genetically overexpressed, can not only mitigate TDP-43-associated neuromuscular dysfunction *in vivo* but also improve TDP-43 cytoplasmic mislocalization in cultured motor neurons. Taken together, our results establish Oxr1 as a novel genetic modifier of TDP-43-related pathology *in vivo*.

## Materials and Methods

### Animals

All experiments were conducted in adherence to the guidelines set forth by the UK Home Office regulations, and with the approval of the University of Oxford Ethical Review Panel. Transgenic TDP-43^M337V^ BAC mice, overexpressing a YPET-tagged TDP-43 protein harbouring the M337V mutation, were maintained on a C57BL/6 J background. Transgenic TDP-43^M337V^ BAC mice are available from The Jackson Laboratory as strain JAX#029266 (https://www.jax.org/strain/029266). Tg (Prnp-Oxr1)/− mice, which were generated by Dr Ben Davies (Wellcome Trust Centre for Human Genetics) as previously described (21), overexpress a HA-tagged Oxr1 transgene driven by the mouse neuronal specific-Prnp promoter (70), and had been maintained on a C57BL/6J background for over 10 generations. For quantitative behavioural and pathological analyses, breeding schemes paired hemizygous TDP-43^M337V/−^ males with hemizygous TDP-43^M337V/−^/Oxr1^Prnp/−^ females to produce litters consisting of control NTg and Oxr1^Prnp/−^ mice, and experimental homozygous TDP-43^M337V/M337V^ and homozygous TDP-43^M337V/M337V^/Oxr1^Prnp/−^ animals. Mice from several litters were required to generate each experimental cohort; for quantitative behavioural and pathological analyses, all cohorts were age and sex-matched. For primary motor neuron cultures, breeding schemes paired homozygous TDP-43^M337V/M337V^ males with Oxr1^Prnp/−^ females to produce litters consisting of hemizygous TDP-43^M337V/−^ and hemizygous TDP-43^M337V/−^/Oxr1^Prnp/−^ mice. The day of plug was designated as E0.5 and the day of birth was designated as postnatal day 0 (P0). Mice received food and water *ad libitum*, and were housed on a 12-h on/off light cycle.

### Behavioural tests

Motor function was tested using a rotarod. In brief, mice were placed on a beam of a rotarod device facing in an orientation opposite to rotation. The rod accelerated from 5 to 40 rpm over a 5-min period and the time latency to fall from the rod (or two full rotations on the rod without an attempt to continue running) was recorded. Mice were tested for one trial each day for three consecutive days at each experimental time point and an average time for the three trials was calculated. To test for muscle strength, mice were placed on a metal mesh grid, which was gently inverted such that the mice gripped on in an upside-down orientation. The grid was held fixed at a height of 40 cm above a horizontal surface and the time latency to fall from the grid was recorded over a 2-min maximum period.

### Histology

For fresh frozen tissue collection, mice were sacrificed by cervical dislocation. For perfused tissue collection, mice were given a lethal intraperitoneal injection of pentobarbitone, followed by transcardial perfusion with 0.9% saline and fixation with 4% paraformaldehyde (PFA). Spinal cords were dissected, post-fixed overnight in 4% PFA at 4°C, cryoprotected with 30% sucrose and mounted in O.C.T. (VWR, Lutterworth, UK) medium. Hind-limb muscle was freshly dissected and frozen in O.C.T. medium on isopentane in liquid nitrogen. Frozen transverse sections were cut on a cryostat (Leica, Newcastle upon Tyne, UK) at 10 μm for muscle and 15 μm for spinal cord. For haematoxylin and eosin stain, muscle sections were air dried and placed in haematoxylin for 10 min, and briefly washed in distilled water. Sections were subsequently bled in 70% ethanol/0.1% hydrochloric acid for 10 s, and washed in distilled water. Sections were then placed in eosin for 2 min, washed in distilled water, dehydrated and mounted. For Nissl staining, spinal cord sections were air dried, fixed in 100% ethanol and hydrated with a series of decreasing alcohol dilutions for 30 s each until washing in distilled water. Sections were subsequently stained for 3 min with 0.5% Cresyl violet (Sigma, Poole, UK.) solution and rinsed in distilled water. Sections were then dehydrated and mounted. For motor neuron counts, all large ventral horn motor neurons on at least 16 matched nissl-stained sections of lumbar spinal cord segments (L1–L6) were counted. For differential ATPase staining, muscle sections were preincubated in 20 mm sodium barbital, 36 mm CaCI_2_, pH 10.3 for 15 min or 50 mm sodium acetate, 30 mm sodium barbital pH 4.2 for 5 min. After rinsing in distilled water, the sections were incubated for 25 min in 20 mm sodium barbital, pH 9.5, containing 9 mm CaCl_2_ and 2.7 mm ATP and then washed in three changes of 1% CaCl_2_ (3 min each). Next, the sections were immersed for 10 min in 2% CoCl_2_ before washing in five changes of 5 mm sodium barbital. After staining with 1% (NH_4_)_2_S for 30 s, the sections were washed with several changes of tap water, and mounted using Aquamount (Life Technologies, Paisley, UK). For quantification, three transverse whole muscle sections at 400 μM intervals were analysed from three to five male animals of each genotype, with adjacent sections used for comparative pH 4.2 and pH 10.3 staining. Images were viewed on a Zeiss Axioskop 2 microscope, acquired using an AxioCam HR digital camera (Zeiss, Cambridge, UK), and processed using AxioVision software (Zeiss).

### Immunohistochemistry

For all immunohistochemistry, perfused tissue sections were subjected to antigen-retrieval by boiling in 0.2% sodium citrate buffer for 20 min in a microwave and washed in ddH_2_O. Endogenous peroxidase activity was first removed by incubating in H_2_O_2_/methanol for 15 min at room temperature (RT) then washing in PBS. Spinal cord sections were blocked in 5% normal goat serum (NGS)/PBS/0.5% Tween-20 at RT for 1 h. For other immunohistochemistry, sections were blocked in 5% NGS/PBS/0.3% Triton-X. Incubation with following primary antibodies and dilutions were conducted overnight at 4°C: rabbit anti-TDP-43 (1:2500, 10 782-2-AP, ProteinTech, Manchester, UK); rabbit anti-GFAP (1:500, ab7260, Abcam, Cambridge, UK); rabbit anti-Iba1 (1:500, 019–19 741, Wako, Osaka, Japan). Sections were then incubated with a biotinylated goat anti-rabbit IgG secondary antibody (Vector Laboratories, Peterborough, UK) at 1:200 for 2 h at RT, washed in PBS three times for 5 min each followed by incubation with an avidin-biotinylated peroxidase complex for 1 h kit). Sections were then washed in PBS three times for 5 min each and then incubated in Impact 3, 3′-diaminobenzidine solution (Vector Laboratories) for 2 min, immediately washed in ddH_2_O for 5 min, dehydrated and mounted using Histomount (National Diagnostics, Atlanta, GA). Images were viewed on a Zeiss Axioskop 2 microscope, acquired using an AxioCam HR digital camera (Zeiss), and processed using AxioVision software (Zeiss). For inflammatory marker quantification, three matched sections from the lumbar spinal cord (L4–5) were processed and quantified in parallel using ImageJ for the total immunopositive area and the number of cells from the number of visible cell bodies.

### Immunoblotting

Tissue extracts were prepared in RIPA buffer (50 mm Tris pH 7.5, 150 mm NaCl, 0.1% SDS, 0.5% Na-deoxycholate, 1% triton) for immunoblotting (IB). Protein concentrations were quantified using BCA assay (Life Technologies). For IB, Laemmli sample buffer was added to 50 μg protein samples and boiled for 5 min, separated on 12% SDS PAGE, and transferred to PVDF membranes (Amersham, Amersham, UK). Membranes were subsequently blocked with 5% skimmed milk in PBS/0.5% Tween-20 (PBST) for 1 h at RT, before incubation with primary antibody overnight at 4°C. The following primary antibodies and dilutions were used: rabbit anti-HA (IB: 1:1000, H6908, Sigma); rabbit anti-Oxr1 (IB: 1:5000/CoIP: 1:250, (71)); rabbit anti-TDP-43 (1:1000, 10 782-2-AP, ProteinTech, Manchester, UK); rabbit anti-GFP (1:1000, A-11122, Invitrogen, Paisley, UK); mouse anti-GAPDH (Glyceraldehyde 3-phosphate dehydrogenase, 1:1000, 649 201 Biolegend, San Diego, CA). After washing with PBST, membranes were incubated with HRP-conjugated anti-rabbit (1:5000, NA934-100UL, Amersham) or anti-mouse (1:5000, NA931-100UL, Amersham) secondary antibodies, and blots were developed using ECL reagents (Amersham). When required, the integrated optical density of each band was measured in ImageJ and expression normalized to GAPDH loading control levels in the same samples for comparative expression assessment.

### Lumbrical muscle dissection and NMJ analysis

Hind-paw lumbrical muscles were dissected as previously described (41). Muscles were permeabilized in 96-well plates with 2% Triton X-100 in PBS for 30 min. After blocking in 4% (w/v) BSA and 1% Triton X-100 in PBS for 30 min, samples were incubated with primary antibodies against neurofilament [2H3, 1:50, supernatant, mouse, developmental studies hybridoma bank (DSHB, Iowa City, IO)] and synaptic vesicles (SV2, 1:100, concentrate, mouse, DSHB) overnight at 4°C in blocking solution. Muscles were washed three times in PBS for 30 min before incubation for 2 h with Alexa Fluor 488 secondary antibody (1:200) and 1.5 μg/ml tetramethylrhodamine α-BTX (BT00012, Cambridge Bioscience, Cambridge, UK) dilution in PBS. Finally, muscles were washed three times for 30 min in PBS, and mounted with Mowiol mounting medium (475 904, Calbiochem, Watford, UK). For denervation analysis, NMJs were viewed using an Axioskop 2 microscope and were scored as being fully innervated (full overlap between the pre-synaptic nerve terminal and the post-synaptic endplate), partially innervated (>80% of regions of the post-synapse lacking neuronal input) or vacant (no pre-synaptic staining overlaying the endplate) as previously described (41,72). A Leica TCS SP5 confocal microscope equipped with 30 mW Argon and HeNe 543 nm lasers and a 63×/1.4NA Plan-Apo oil immersion objective was used to image representative examples of NMJ denervation and also NMJs for endplate analysis. Z-stacks were projected into single images using Leica Application Suite software and the resulting ‘collapsed’ images were used for all analyses. Only NMJs with clearly visible presynaptic axons were scored. To measure NMJ area, the perimeter circumscribing the postsynaptic staining from ‘collapsed’ Z-stack images (24 sections, 0.5 μm step size intervals, 11.6 μm total stack depth) was drawn by hand and the enclosed surface area measured using ImageJ as performed previously (41). ≥57 NMJs were scored per mouse for denervation analysis and 30–50 NMJs per mouse were scored for NMJ endplate analysis.

### Primary motor neuron culture and immunocytochemistry

To culture primary motor neurons, whole spinal cords were dissected from TDP-43^M337V/−^ and TDP-43^M337V/-^Oxr1^Prnp/−^ embryos at E13.5. Spinal cords were digested in trypsin solution (Gibco) for 10 min at 37°C, followed by incubation with trypsin inhibitor (0.8 mg/ml; Sigma) for 5 min at RT. Spinal cords were then transferred to motor neuron growth medium (MNGM) that consisted of Neurobasal medium (Invitrogen, Paisley, UK) supplemented with 10% horse serum (heat inactivated; Invitrogen), 1% B27 supplement (Invitrogen) and 1% Glutamax I (Invitrogen). After cell trituration, the dissociated spinal cord cell suspension was subsequently passed through a 40 μm cell strainer. Purification of motor neurons was performed using Optiprep density 1.32 (Sigma) in MNGM and density gradient centrifugation, as described previously (64). After washing motor neurons in fresh MNGM, motor neurons were seeded at a density of 20 000 cells/cm^2^ on sterilized glass coverslips (Life Technologies) pre-coated in poly-L-ornithine (Sigma). Motor neurons were allowed to attach in a humidified 37°C/5% CO_2_ incubator for 1 h, before MNGM was replaced with fresh MNGM supplemented with 10 ng/ml brain derived neurotrophic factor (PeproTech, London, UK), glial-cell derived neurotrophic factor (PeproTech), neurotrophin-3 (PeproTech) and 25 ng/ml of ciliary neurotrophic factor (PeproTech). Cultured motor neurons were allowed to expand for 7 days *in vitro* (DIV) prior to assaying.

For immunocytochemistry, motor neuron cultures were fixed in 4% PFA in PBS (pH 7.2) for 10 min. Motor neurons were then washed in PBS three times for 15 min, and blocked and permeabilized for 1 h in PBS containing 5% NGS and 0.5% Triton X-100. Incubation with the following primary antibodies and dilutions was conducted overnight at 4°C: mouse anti-SMI32 (1:1000: 801 701, Biolegend); rabbit anti-GFP (1:200: A-11122, Invitrogen). After washing in PBS, coverslips were incubated with AlexaFluor secondary antibodies for 2 h at RT. After three washes in PBS, motor neurons were mounted with DAPI-containing Vectashield mounting medium. Cultures were imaged using a Leica TCS SP5 confocal microscope. All Images were processed in ImageJ and only cells positive for SMI32 were further analysed. To calculate cytoplasmic localization of YPET-tagged human TDP-43^M337V^, the perimeters circumscribing both the external membrane of the cytoplasm of the entire neuron and the nucleus were drawn by hand and GFP fluorescence intensity was recorded in both areas of the cell. The proportion of TDP-43 cytoplasmic localization was calculated as: (intensity of cytoplasmic signal/intensity of nuclear and cytoplasmic signal). Between 45 and 60 motor neurons were used for protein localization analysis per individual embryonic motor neuron preparation. Cultures from six embryos from at least two independent litters were analysed per genotype.

### Statistical analysis

Results were analysed using Prism (GraphPad Software, Inc. San Diego, CA) with the statistical tests as described in the figure legends. Data are presented as mean ± SEM (standard error of mean), with *N* indicating the number of independent biological replicates used in each group for comparison. For all quantitative data, experimenters were blinded to the genotypes. To determine the sample sizes used for each experiment, power calculations were carried out (73) using our own datasets of the same type (stated as: standard deviation/target effect size given as percentage difference between experimental groups/sample size using a significance value of *P* < 0.05 and power level of 80%). Figure 1B: 6/20/*N* = 3; Figure 2A-B: 20/15/*N* = 6; Figure 2C and D: 20/25/*N* = 6; Figure 2E and F: 2/10/*N* = 5; Figure 3: 5/15/*N* = 3; Figure 4: 50/20/*N* = 3; Figure 5A and B: 1/40/*N* = 3; Figure 6B: 2/25/*N* = 3; Figure 6C and D: 0.3/50/*N* = 3.

## Supplementary Material

Suppl_Fig_1_02_07_19_ddz190

Suppl_Fig_2_02_07_19_ddz190

Suppl_Fig_3_02_07_19_ddz190

Williamson_et_al_Suppl_legends_and_table_02_07_19a_ddz190
