# Supplementary figures and images for: Neuronal over-expression of Oxr1 is protective against ALS-associated mutant TDP-43 mislocalisation in motor neurons and neuromuscular defects in vivo
Source: Hum Mol Genet. 2019 Sep 6;28(21):3584–99. doi: 10.1093/hmg/ddz190 (PMC6927465; doi:10.1093/hmg/ddz190)

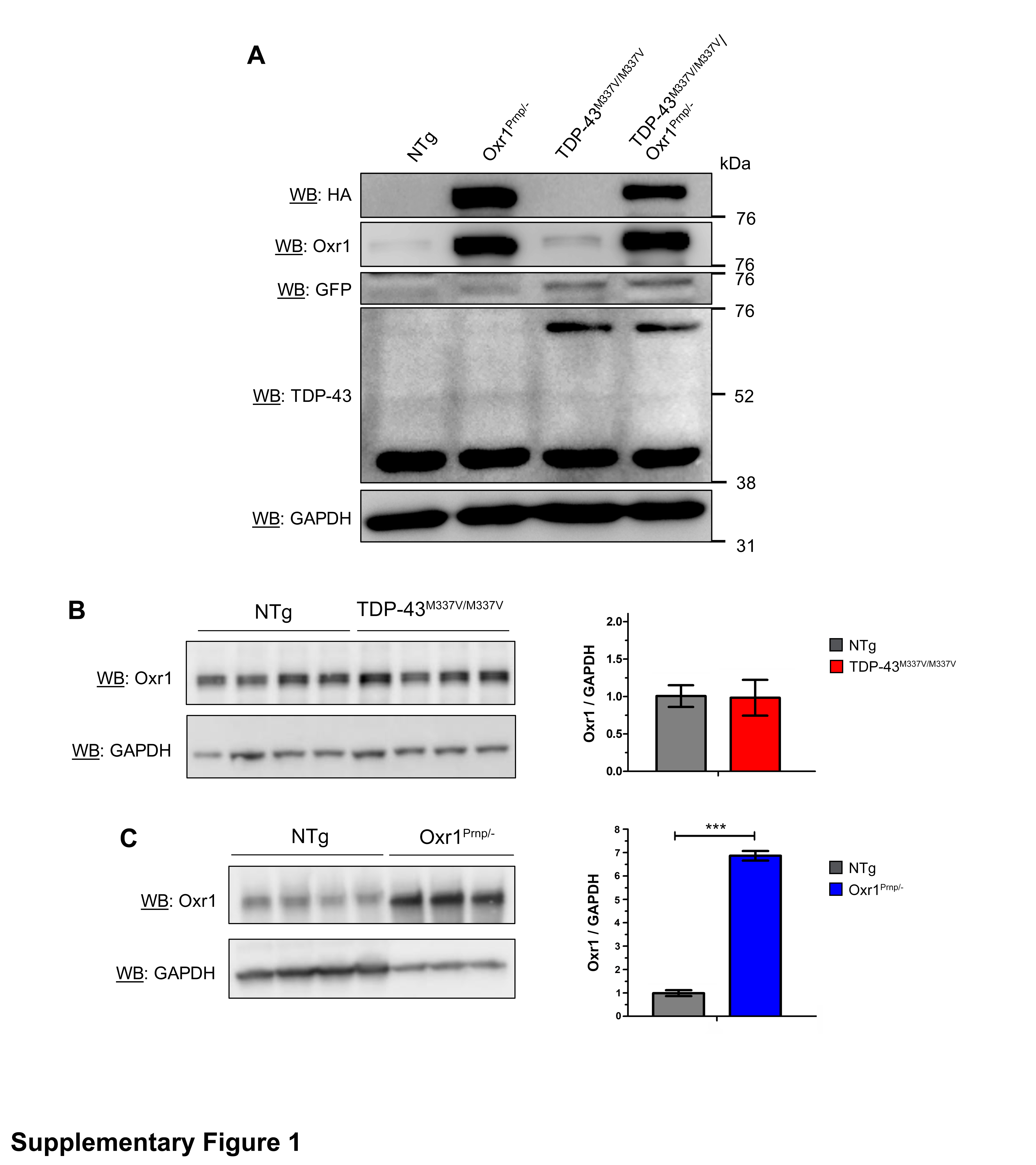

Supplement: Suppl_Fig_1_02_07_19_ddz190 [file suppl_fig_1_02_07_19_ddz190.png]

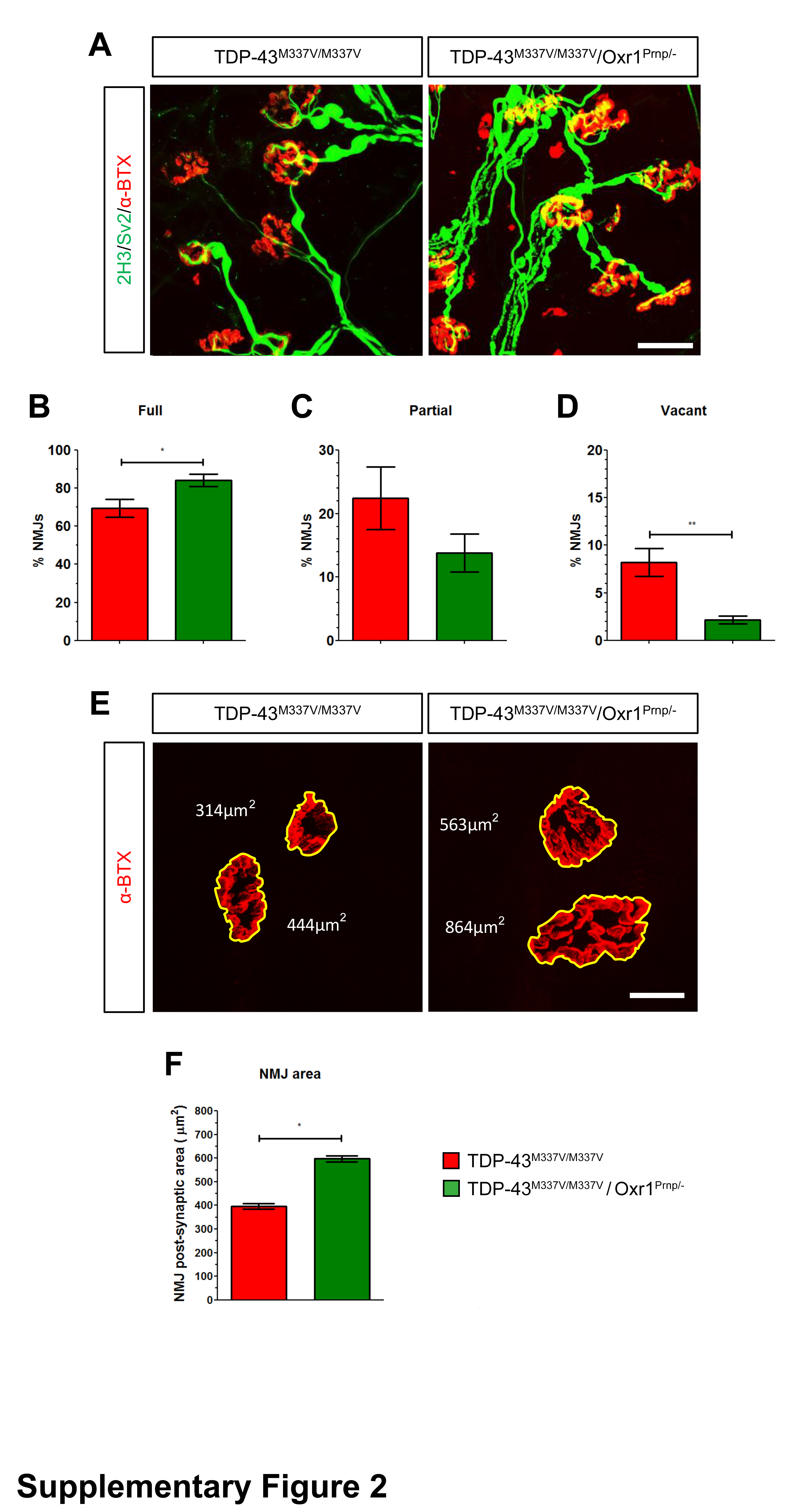

Supplement: Suppl_Fig_2_02_07_19_ddz190 [file suppl_fig_2_02_07_19_ddz190.zip › Suppl_Fig_2_02_07_19_ddz190.tif]

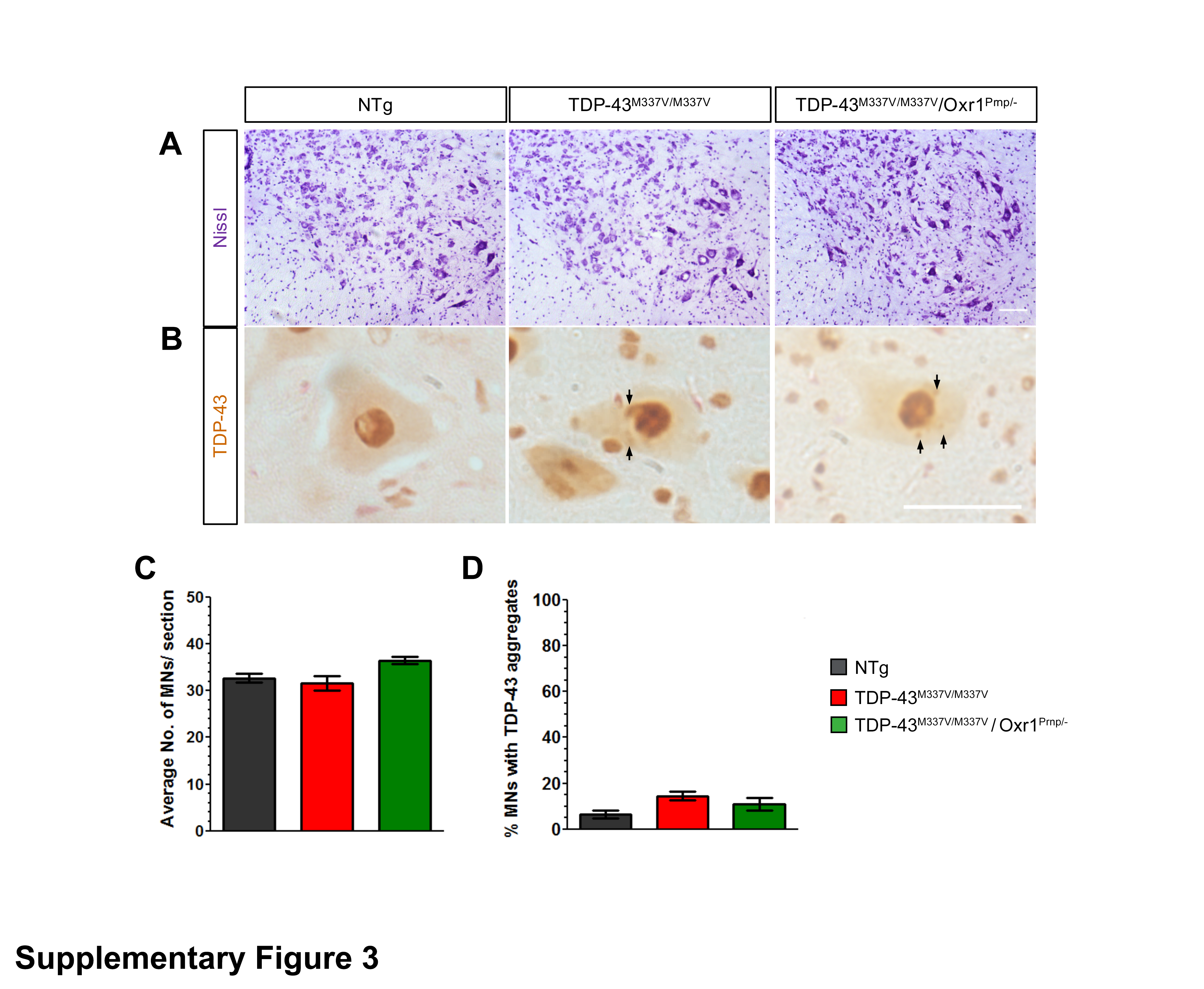

Supplement: Suppl_Fig_3_02_07_19_ddz190 [file suppl_fig_3_02_07_19_ddz190.zip › Suppl_Fig_3_02_07_19_ddz190.tif]
