# Supplementary material for: Neuronal over-expression of Oxr1 is protective against ALS-associated mutant TDP-43 mislocalisation in motor neurons and neuromuscular defects in vivo
Source: Hum Mol Genet. 2019 Sep 6;28(21):3584–99. doi: 10.1093/hmg/ddz190 (PMC6927465; doi:10.1093/hmg/ddz190)
Supplement: Williamson_et_al_Suppl_legends_and_table_02_07_19a_ddz190 [file williamson_et_al_suppl_legends_and_table_02_07_19a_ddz190.docx]

**Williamson et al.**

**Supplementary figures legends and tables**

**Supplementary Figure 1: Expression of human TDP-43 and Oxr1 transgenes in the spinal cord. (A)** Representative Western blot of the HA-tagged Oxr1 transgene, endogenous Oxr1, human Ypet-tagged TDP-43^M337V^ (at approximatley 70 kDa) and endogenous mouse TDP-43 (at approximatley 43 kDa) from whole mouse spinal cord samples at 18 months of age from the genotypes indicated. **(B)** Western blot quantification of endogenous Oxr1 expression in NTg and TDP-43^M337V/M337V^ homozygous spinal cord at 18 months of age. **(C)** Western blot quantification of Oxr1 over-expression in Oxr1^Prnp/-^ spinal cord at 18 months versus NTg controls. GAPDH was used as a loading control with 50μg protein loaded in each lane. (***p<0.001; unpaired t-test).

**Supplementary Figure 2: Overexpression of Oxr1 improves muscle denervation and NMJ degeneration in female TDP-43^M337V/M337V^** **mutants. (A)** Representative images showing lumbrical NMJs from female TDP-43^M337V/M337V^ and TDP-43^M337V/M337V^/Oxr1^Prnp/-^ mice at 18 months of age. Levels of denervation were assessed by determining the overlap between pre-synaptic (2H3/SV2) and post-synaptic (α-BTX) staining. (**B-D**) Denervation analysis reveals a significant decrease in fully innervated and a significant increase in partially innervated and vacant NMJs in TDP-43^M337V/M337V^ mice, which is rescued by overexpression of Oxr1. (**E**) Representative images of motor endplates used to measure post-synaptic area. (**F**) Motor endplate area is reduced in TDP-43^M337V/M337V^ mice, and is rescued by overexpression of Oxr1. Values are shown as the mean ± SEM. N=3-6 per genotype, N=30-50 NMJs per animal. (*p<0.05, **p<0.01, ***p<0.001; One-way ANOVA with one-way ANOVA with Dunnett’s multiple comparison test). Scale bars: 20µm.

**Supplementary Figure 3: No motor neuron loss or cytoplasmic aggregation of TDP-43 in spinal cord of male TDP-43^M337V/M337V^ mice. (A)** Representative images of Nissl-stained spinal cord sections and **(B)** spinal motor neurons stained with anti-TDP-43 from NTg, TDP-43^M337V/M337V^ and TDP-43^M337V/M337V^/Oxr1^Prnp/-^ mice. **(C)** Motor neuron counts from Nissl-stained spinal cord sections (16 sections per animal) reveal no motor neuron loss in TDP-43^M337V/M337V^ mice compared to NTg littermates. **(D)** Motor neuron counts containing TDP-43 immunopositive cytoplasmic aggregates (arrows) reveal no significant increase in motor neurons from TDP-43^M337V/M337V^ mice compared to NTg littermates. Values are shown as the mean ± SEM. **(C)** N=4, **(D)** N=3-4, one-way ANOVA with one-way ANOVA with Dunnett’s multiple comparison test. (**A**) Scale bar: 100µm (**B**) Scale bar: 50µm.





**Supplementary Table 1: Numbers of mice used for each motor function testing cohort**
